# Interfering with CXCR4 expression inhibits proliferation, adhesion and migration of breast cancer MDA-MB-231 cells

**DOI:** 10.3892/ol.2014.2323

**Published:** 2014-07-07

**Authors:** SHANYU GUO, DAN XIAO, HUIHUI LIU, XIAO ZHENG, LEI LIU, SHOUGUI LIU

**Affiliations:** Department of General Surgery, Shanghai Ninth People’s Hospital, Shanghai Jiaotong University School of Medicine, Shanghai 200011, P.R. China

**Keywords:** CXC chemokine receptor 4, RNA interference, breast cancer

## Abstract

To investigate the effect and mechanism of the CXC chemokine receptor 4 (CXCR4) in the proliferation and migration of breast cancer, a short-hairpin RNA (shRNA) eukaryotic expression vector targeting CXCR4 was constructed, and the impact of such on the proliferation, adhesion and migration of human breast cancer MDA-MB-231 cells was observed. The fragments of CXCR4-shRNA were synthesized and cloned into a pGCsi-U6-Neo-green fluorescent protein vector. The recombinant plasmids were transfected into 293T cells and the most efficacious interfering vector was selected. MDA-MB-231 cells were transfected by liposome assay. The effects of silencing CXCR4 expression by shRNA on the growth, adhesion and migration of MDA-MB-231 cells were determined by Cell Counting Kit-8, cell-matrix adhesion and wound-healing assays. The shRNA eukaryotic expression vectors targeting CXCR4 (CXCR4-shRNA) were successfully constructed and transfected into 293T cells. Quantitative polymerase chain reaction and western blot analysis revealed that the maximum inhibitory rate of CXCR4 expression was 81.3%. CXCR4-shRNA transfection significantly inhibited the proliferation of MDA-MB-231 cells (P<0.05), as well as the adhesion between MDA-MB-231 cells and the extracellular matrix (P<0.05). Furthermore, wound-healing assays demonstrated that the migration distance of MDA-MB-231 cells in the CXCR4-shRNA transfection group was significantly smaller than that in the control plasmid and blank control groups (P<0.01). The CXCR4-shRNA interfering vector specifically inhibited CXCR4 expression, as well as the proliferation, adhesion and migration of MDA-MB-231 cells.

## Introduction

Breast cancer is the leading cause of cancer-associated mortality among females worldwide. Due to the progress achieved in the treatment of this type of cancer, patient mortality is now increasingly associated with the occurrence of distant metastases (1). Certain organs are favored sites for circulating cancer cells to develop metastases, which occur as a result of a permissive microenvironment in the target tissue that facilitates tumor growth (2). Breast cancer is characterized by a distinct metastatic pattern involving the regional lymph nodes, bone marrow, lung and liver.

Tumor cell migration and metastasis is a complex process, which is regulated by chemokines and their receptors (3). Chemokines are a superfamily of small, low molecular weight proteins that induce cytoskeletal rearrangement, firm adhesion to endothelial cells and directional migration, via their interaction with G-protein-coupled serpentine receptors (4). The CXC chemokine receptor 4 (CXCR4), a member of the G-protein-coupled receptor superfamily, and is the only physiological receptor of high specificity for stromal cell derived factor-1 (SDF-1). It is closely involved and important in various physiological and pathological processes *in vivo*, including immune defense and anti-inflammation. Previous studies (5–8) have indicated that CXCR4 is expressed in a number of tumor cells, and its specific binding with SDF-1 in certain tissues is key for tumor genesis, progression and metastasis.

In the present study, a short-hairpin RNA (shRNA) eukaryotic expression vector targeting CXCR4 was constructed and transfected into 293T cells *in vitro.* The most efficacious interfering vector was selected and transfected into the highly invasive breast cancer MDA-MB-231 cell line, and the effect of silencing the CXCR4 gene on the proliferation, adhesion and migration of 231 cells was observed *in vitro*. The results may provide a basis for cancer therapy which targets CXCR4.

## Materials and methods

### Reagents

Human renal embryonic 293T cells were obtained from Shanghai Tongji University (Shanghai, China). The breast cancer MDA-MB-231 cell line was purchased from the Chinese Academy of Sciences (Shanghai, China). PGCsi-U6-Neo-green fluorescent protein (GFP) vacant plasmids were obtained from Shanghai Jiaotong University (Shanghai, China) and DH5α competent cells were purchased from Shanghai Hi-tech Bioengineering Co. Ltd. (Shanghai, China). Fetal bovine serum (FBS) and high-glucose Dulbecco’s modified Eagle’s medium were purchased from Gibco-BRL (Carlsbad, CA, USA). RPMI-1640 medium was purchased from Nanjing KeyGen BioTech Co. Ltd. (Nanjing, China). The incision enzymes, *Bam*HI, *Hin*dIII, *Nhe*I and T4DNA ligase were all purchased from Fementas Life Sciences (Rockford, IL, USA). Lipofectamine 2000 was purchased from Invitrogen Life Technologies (Carlsbad, CA, USA). RNA TRIzol reagent and rabbit polyclonal anti-human CXCR4 multi-clone antibody (1:250) were purchased from Santa Cruz Biotechnology, Inc. (Santa Cruz, CA, USA). A reverse transcription reagent kit was purchased from Takara Bio, Inc. (Shiga, Japan). Cell Counting Kit-8 (CCK-8) was purchased from Beyotime Biotech. (Jiangsu, Japan) and Matrigel was purchased from BD Biosciences (Franklin Lakes, NJ, USA).

### Construction of plasmid vectors

According to the restriction endonuclease digestion site of the pGCsi-U6-Neo-GFP vacant plasmid, two interfering sequences of CXCR4 shRNA and a negative control sequence (Table I) were selected for the construction of DNA and primers, which were produced by Shanghai Hi-tech Bioengineering Co. Ltd. Reverse transcription-polymerase chain reaction (RT-PCR) and western blot analysis were used to screen the pairs to identify those exhibiting the most efficacious interference.

### Recombinant plasmids

Sense and anti-sense primers (2 μl, respectively) were mixed with 2 μl annealing buffer and 4 μl double-distilled water to produce a combined double strand, which was annealed from 95 to 25°C at a velocity of 0.5°C/sec for 2 min. The restriction endonucleases, *Bam*HI and *Hin*dIII, were used to digest PGCsi-U6-Neo-GFP vacant plasmids at 37°C in a water bath for 3 h. Agarose gel electrophoresis was used to analyze and retrieve the linear products. A total of 1 μl diluted annealing primer was then conjugated with the linear vector in the ratio of 4:1 at 22°C for 1 h, then transfected to the competent bacteria DH5α. Next, the bacteria solution was added to the LB agar plate with ampicillin (Hubei Shengtian Hengchuang Biotechnology Co., Ltd., Shanghai, China) at 37°C overnight. The selected monoclonal bacterial colony was then amplified for plasmid extraction and *Nhe*I endonuclease digestion. The products were analyzed by agarose gel electrophoresis and DNA sequencing was performed by Shanghai Hi-tech Bioengineering Co. Ltd to identify successful recombinants. The amplified recombinant vectors were maintained at −20°C.

### Selection of shRNA eukaryotic expression vectors targeting CXCR4

293T cells at the logarithmic phase were seeded in six-well culture plates for 24 h and transfected with recombinant plasmids by Lipofectamine 2000, when the cells reached ~70% confluence. GFP expression was observed under a fluorescence microscope (DM3000; Leica, Mannheim, Germany) at 24, 48 and 72 h following transfection, to calculate the transfection efficiency of the plasmids.

### Expression of CXCR4 mRNA in 293T cells by RT-PCR

A total of 2 μl mRNA was extracted from 293T cells using TRIzol reagent 48 h following transfection. The mRNA was then reversely transcribed to cDNA using the reverse transcription kit (Takara bio, Inc.) at 37°C for 15 min, and amplified by PCR for 5 min. The primer sequences were as follows: Forward, 5′-GAC AGG ATG CAG AAG GAG ATT ACT-3′ and reverse, 5′-TGA TCC ACA TCT GCT GGA AGG T-3′ for β-actin, yielding a 318-bp amplified fragment; forward, 5′-GGA GGC TGG CAA CAT AAC-3′ and reverse, 5′-TGG CAG GGA ACG TCT AAT-3′ for CXCR4, yielding a 227-bp amplified fragment. The reaction procedure was as follows: 94°C for 3 min, 94°C for 30 sec, 59°C for 30 sec, 72°C for 1 min and 72°C for 5 min, for 30 cycles. Following electrophoresis, the images were scanned and analyzed by Gel-Pro Analyzer (Media Cybernetics, Silver Spring, MD, USA) to determine the integrated optical density (OD) and analyzed using gray values to calculate interference efficiency following 1% agarose gel electrophoresis.

### Western blot analysis of CXCR4 protein expression in 293T cells

Total protein of 293T cells was extracted by radioimmunoprecipitation assay buffer and phenylmethanesulfonyl fluoride 72 h following transfection, and the concentration of CXCR4 protein was detected using the bicinchoninic acid assay method. A total of 50 μg extracted protein solution was mixed with sample buffer for denaturation at 99°C for 10 min to perform sodium dodecyl sulfate-polyacrylamide gel electrophoresis, whereby the protein was transferred from gelatin to a polyvinylidene fluoride membrane, and blocked with non-fat milk for 2 h. Primary antibody (CXCR4, 1:200; β-actin, 1:500) was added at 4°C and left overnight. The secondary antibody (CXCR4, 1:10,000, β-actin, 1:10,000) was added the next day at room temperature for 2 h incubation and visualized using the EZ-ECL chemiluminescence detection kit for HRP (Bioind, Kibbutz Beit Haemek, Israel). The following groups were used: 1, pGCsi-CXCR4-1/shRNA; 2, pGCsi-CXCR4-2/shRNA; 3, negative control (blank plasmid); and 4, untreated group (untransfected plasmid).

### Transfection of MDA-MB-231 cells with shRNA interfering vector

The most efficacious interfering vector was selected to transfect MDA-MB-231 cells for two days according to the results of RT-PCR and western blot analysis. Medium supplemented with G418 (500 mg/l; Gibco-BRL) was added for cell screening. After 10–14 days, monoclonal resistant cells were obtained using limiting dilution assay and maintained in medium supplemented with G418 (200 mg/l) for amplification.

### Assessment of proliferation of MDA-MB-231 cells with silenced CXCR4 by CCK-8 assay

MDA-MB-231 cells were seeded in 96-well culture plates at a concentration of 1×10^5^ cells/ml. Following 24 h incubation, 100 μl medium was removed from each well and 10 μl CCK-8 was added and incubated for 1 h at 37°C. Absorbance was measured using an ultraviolet spectrophotometer (UV-5100; Shanghai Yuanxi Instrument Co., Ltd., Shanghai, China) at a wavelength of 450 nm for five days. The results were calculated as the mean values of five wells for each group, and the assay was performed in triplicate.

### Measurement of MDA-MB-231 cell adhesion by cell-matrix adhesion assays

Fibronectin (FN; Phoenix Pharmaceuticals Inc., Burlingame, CA, USA) was used to simulate an extracellular matrix environment and bovine serum albumin (BSA), simulating basement membrane, was used as the control. A total of 20 mg/l FN and 10 g/l BSA (Shenzhen Niubang Bio-technology Company, Shenzhen, China) coated the 96-well culture plates (50 μl/well), which were air dried on a sterilized bench and stored at 4°C until use. The cells were hydrated using phosphate-buffered saline, and 50 μl serum-free BSA medium (10 g/l) was added to each well at 37°C for 30 min. The cells were then collected at the logarithmic phase and RPMI-1640 medium supplemented with 0.1% BSA was added, adjusting the concentration to 1×10^5^ cells/ml. Next, 100 μl of the previous cell suspension was added to each well and incubated at 37°C. A total of 90 μl serum-free medium and 10 μl CCK-8 reagent was then added and cells were incubated at 37°C for 1 h, following discarding former medium, at 30, 60 and 90 min, respectively. The cells were then washed with serum-free medium three times to remove floating cells. OD was measured at a wavelength of 450 nm. The cell adhesion of each group was calculated according to the OD value of the BSA group, using the following formula: Cell adhesion (%) = OD value of cells in experimental group/OD value of BSA group × 100. The results were calculated as the mean values of six wells per group and the experiment was performed in triplicate.

### Measurement of MDA-MB-231 cell migration by wound-healing assays

MDA-MB-231 cells from each group were seeded in six-well culture plates at a concentration of 2×10^6^ cells/ml. A scratch in the middle of the wells was established by a cell knife when cells reached 80% confluence, and the corresponding positions relative to the wound zone were observed under a microscope (Olympus BH2-MJLT; Olympus Corporation, Shanghai, China). Cells were washed three times with serum-free medium and antibiotic-free RPMI-1640 medium supplemented with 10% FBS, and then cultured for 24 h. Images were captured using an inverted microscope [XploRA INV; HORIBA (China) Trading Co., Ltd., Shanghai, China]. Four marks of equidistance along the scratch were established as assay points, and the actual migration of cells was calculated as the mean values according to the distance between the original wound zone and marks. Experiments were repeated three times.

### Statistical analysis

Statistical significance was assessed by comparing the mean ± standard deviation values using the Student’s t-test for independent groups. P<0.05 was considered to indicate a statistically significant difference.

## Results

### Identification of recombinant CXCR4

**Figure 1:**
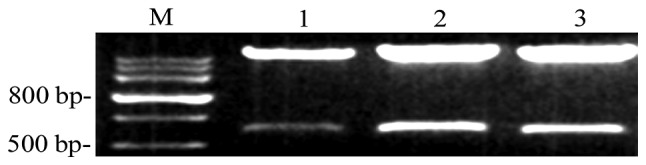
Identification of recombinant CXCR4 interfering RNA plasmid by restriction enzyme digestion. M, marker; 1, pGCsi-CXCR4-1/ShRNA; 2, pGCsi-CXCR4-2/ShRNA; and 3, negative control. CXCR4, CXC chemokine receptor 4.

Interfering RNA vectors were digested using restriction endonucleases and agarose gel electrophoresis was performed. It was found that recombinant plasmids exhibited two DNA fragments of 5,600 and 650 bp following digestion. The fragments of CXCR4 shRNA were as expected and were identical to the designed sequences (Fig. 1).

### Transfection efficiency of recombinant plasmids in 293 T cells

**Figure 2:**
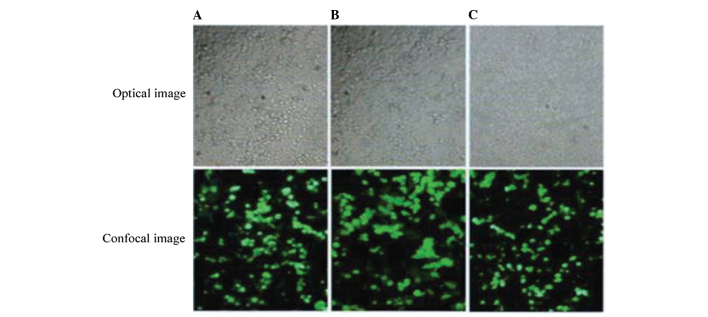
Transfection efficiency of interfering RNA CXCR4 vector in 293T cells (magnification, ×100). (A) pGCsi-CXCR4-1/ShRNA, (B) pGCsi-CXCR4-2/ShRNA and (C) negative control. CXCR4, CXC chemokine receptor 4.

A total of 293T cells were transfected with each group of plasmids and observed under an inverted microscope 24, 48 and 72 h following transfection. The light of highest intensity was green fluorescence following 72 h transfection with a transfection efficiency of ~90% (Fig. 2).

### mRNA expression of CXCR4 in 293T cells

The relative expression levels of CXCR4 mRNA 48 h following transfection were 1.13±0.19, 0.30±0.09, 1.28±0.11 and 1.60±0.61 for the pGCsi-CXCR4-1/ShRNA, pGCsi-CXCR4-2/ShRNA, negative control group and blank control groups, respectively (data not shown). No significant difference was identified between the negative and control groups, indicating that no RNA interference of CXCR4 mRNA occurred in cells of the negative control group (P>0.05). However, a statistically significant difference in inhibitory rate was identified between pGCsi-CXCR4-2/ShRNA (81.3%) when compared with the negative and blank control groups (P<0.05).

### CXCR4 protein expression in 293T cells

**Figure 3:**
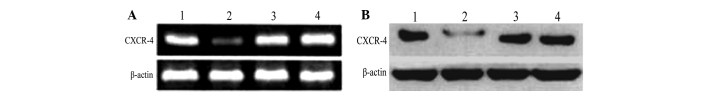
(A) mRNA and (B) protein expression of CXCR-4 in 239T cells by RNA interference. 1, pGCsi-CXCR4-1/ShRNA; 2, pGCsi-CXCR4-2/ShRNA; 3, negative control; and 4, blank control. CXCR4, CXC chemokine receptor 4.

RT-PCR analysis of the protein expression of CXCR4 in each group 72 h following transfection demonstrated that CXCR4 protein expression in the pGCsi-CXCR4-2/ShRNA group was the lowest and no significant differences were identified between the negative and blank control groups (Fig. 3).

### Inhibition of proliferation of MDA-MB-231 cells by CXCR4 silencing

**Figure 4:**
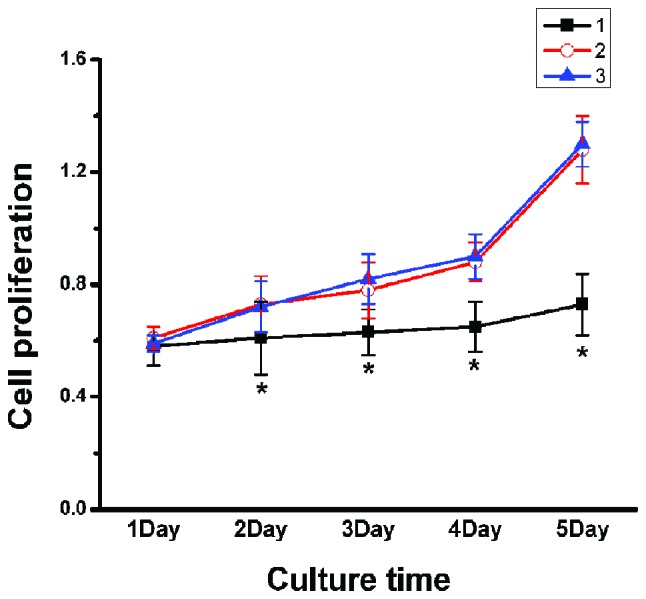
Silencing CXCR4 expression inhibits the proliferation of MDA-MB-231 cells. ^*^P<0.05, vs. negative control or blank control. 1, pGCsi-CXCR4-1/ShRNA; 2, negative control; and 3, blank control. CXCR4, CXC chemokine receptor 4.

A statistically significant difference was identified between the CXCR4-shRNA group and blank control group, as well as the negative control group (P<0.05); however, no significant difference was identified between the negative and blank control groups. Therefore, these results suggest that the proliferation of MDA-MB-231 cells may be significantly inhibited by RNA interference, which targets CXCR4 gene expression (Fig. 4).

### Inhibition of MDA-MB-231 cell adhesion by CXCR4 silencing

A significant decrease in the number of adhesive cells in the CXCR4-shRNA transfection group was observed when compared with the negative and blank control groups (P<0.05); however, no significant difference was identified between the negative and blank control groups (P>0.05), which indicated that breast cancer cells may be inhibited by the downregulation of the CXCR4 gene *in vitro* (Table II).

### Inhibition of MDA-MB-231 cell migration by CXCR4 silencing

Wound-healing assays revealed that the migration distance of MDA-MB-231 cells in the CXCR4-shRNA transfection group (0.42±0.09 mm) was significantly smaller than that in the control plasmid (2.16±0.44 mm) and the blank control (2.38±0.56 mm) groups (P<0.01), which indicated that the downregulation of the CXCR4 gene may significantly inhibit the migration of breast cancer cells.

## Discussion

Breast cancer is one of the most common types of malignant tumors among females, and the incidence is increasing rapidly in China. Disease recurrence and metastasis are key points in the prognosis of breast cancer. Cancer metastasis is a complex process in which malignant cells break away from the primary tumor, attach to the degraded proteins of the surrounding extracellular matrix and migrate to other locations via the bloodstream or the lymphatic system. Tumor cell proliferation, adhesion and migration are involved and are tightly regulated during the metastatic process (9). Various factors are involved in tumor recurrence and metastasis, including modification of cell gene regulation, disorder of cytokine secretion, enhancement of tumor immunological tolerance, degradation of extracellular matrix and inhibition of adhesion. CXCR4, a highly conserved G-protein-coupled receptor (10) of high specificity to SDF-1, is coded for by a 352-amino acid protein with seven transmembrane domains. The CXCR4 gene was originally separated and purified from human monocytes and was found to be located at human chromosome 2q21. Previous studies (11) have predominantly focused on its critical function as an essential coreceptor of the CD4 molecule, which allows the HIV virus to enter T cells and diffuse *in vivo*. CXCR4 is expressed in various tumor cells (12–14), and the CXCR4/SDF-1 axis has a significant role in malignant tumor genesis, adhesion, infiltration and metastasis (15–17). The CXCR4/CXCL12-axis has been demonstrated to exhibit a critical role in the trafficking and homing of normal stem cells and metastasis of cancer stem cells to organs that express high levels of CXCL12, including the lymph nodes, lungs, liver and bone (17). Smith *et al* (18) revealed that metastasis of breast cancer cells in mice lungs was delayed by intravenous injection at the caudal vein with AMD 3100, a CXCR4 antagonist.

The RNAi technique is a gene therapy method which uses small interfering double-stranded RNA, originating from the inside of the cell or transfection to perform gene silencing following transcription. This process of special homologous mRNA degradation is mediated by double-stranded RNA (19–21). Therefore, RNAi may block tumor-associated gene expression and effectively terminate the translation process of target proteins to treat tumors (22). In the present study, CXCR4 mRNA was silenced and CXCR4 protein expression was decreased using the hairpin structure shRNA technique to specifically degrade relevant sequences. This allowed the selection of the most effective interfering CXCR4-shRNA sequence, which had an inhibitory rate of 81.3%, which was statistically significant when compared with the blank and negative control groups.

To further investigate the impact of silencing the CXCR4 gene on the malignant bionomics of breast cancer cells, MDA-MB-231 cells were transfected with selected sequences of CXCR4-shRNA, and the CCK-8 kit, cell adhesion assays and wound-healing assays were used to determine changes prior to and following shRNA interference. A CCK-8 assay was performed to determine the proliferation of tumor cells *in vitro* and the result revealed that compared with the negative and blank control groups, the proliferation of CXCR4-shRNA was significantly inhibited (P<0.05). These results indicated that CXCR4 exhibits a critical function in tumor genesis and proliferation of breast cancer cells, and that the selected vector inhibited breast cancer cell proliferation *in vitro,* which was consistent with the results of Lapteva *et al* (23).

Adhesion, degradation and removal are the three predominant stages of tumor migration and infiltration. Ueda *et al* (24) demonstrated that the binding between CXCR4 and its ligand, SDF-1, downregulated the expression of E-cadherin and inhibited adhesion between tumor cells to promote metastasis, which was inhibited by an anti-CXCR4 monoclonal antibody (25). Furthermore, Zeelenberg *et al* (26) revealed that SDF-1/CXCR4 evoked the aggregation and re-distribution of cytoskeletal proteins to regulate cell movement and migration. In addition, the migration of breast cancer cells was inhibited by a CXCR4 antagonist, as well as infiltration to the lungs and bone (27,28). In this study, cell-matrix adhesion and wound-healing assays were performed to determine the impact of CXCR4-shRNA on cancer cell adhesion and migration. The results indicated that blockade of CXCR4 expression significantly inhibited tumor cell migration and adhesion to the matrix (P<0.05), indicating that the upregulation of CXCR4 causes epithelial cell instability and promotes tumor cell migration and infiltration.

In conclusion, in this study shRNA eukaryotic expression vectors targeting CXCR4 were successfully constructed, and CXCR4-shRNA was found to significantly inhibit the proliferation, adhesion and migration of breast cancer cells *in vitro*. These results may provide a foundation for further study regarding the mechanisms of CXCR4 involved in breast cancer growth and metastasis.

## Figures and Tables

**Table I:** Sequences of interference RNA used in the study.

Gene	Target sequence	ShRNA sequences
CXCR4-1	TCCTGGCCTTCATCAGTCT (6)	5′-GAT CCT CCT GGC CTT CAT CAG TCT TTC AAG AGA AGA CTG ATG AAG GCC AGG ATT TTT GGA AGC TAG GA-3′
CXCR4-2	TGCCCACCATCTACTCCAT (7)	5′-GAT CCT GCC CAC CAT CTA CTC CAT TTC AAG AGA ATG GAG TAG ATG GTG GGC ATT TTT GGA AGC TAG CA-3′
Negative control	AATCGCATAGCGTATGCCGTT (8)	5′-GAT CCA ATC GCA TAG CGT ATG CCG TTT TCA AGA GAA ACG GCA TAC GCT ATG CGA TTT TTT TGG AAG CTA GCA-3′

CXCR4, CXC chemokine receptor 4.

**Table II:** Silencing of CXCR4 expression inhibited the adhesion of MDA-MB-231 cells.

Group	30 min	60 min	90 min
PGCsiCXCR4 1–2/ShRNA	5.97±2.41^a^	6.64±2.80^a^	7.81±0.77^a^
Negative control	12.04±3.31	13.43±2.50	14.51±5.44
Blank control	12.42±3.49	14.37±1.73	15.49±1.38

a P<0.05, vs. negative control or blank control.

CXCR4, CXC chemokine receptor 4; shRNA, short-hairpin RNA.
